# Chloroplast clustering around the nucleus induced by OMP24 overexpression unexpectedly promoted PSTVd infection in *Nicotiana benthamiana*

**DOI:** 10.1111/mpp.13385

**Published:** 2023-09-11

**Authors:** Kelei Han, Zhaoxing Jia, Yuhong Zhang, Huijie Zhou, Shan Bu, Jianping Chen, Dankan Yan, Rende Qi, Fei Yan, Jian Wu

**Affiliations:** ^1^ State Key Laboratory for Managing Biotic and Chemical Threats to the Quality and Safety of Agroproducts Institute of Plant Virology, Ningbo University Ningbo China; ^2^ Key Laboratory of Biotechnology in Plant Protection of MARA and Zhejiang Province Institute of Plant Virology, Ningbo University Ningbo China; ^3^ Institute of Plant Protection and Agro‐Products Safety, Anhui Academy of Agricultural Sciences Hefei China

**Keywords:** chloroplast outer membrane protein 24, H_2_O_2_, perinuclear chloroplast clustering, potato spindle tuber viroid

## Abstract

Chloroplast clustering around the nucleus is a well‐known mechanism that occurs in response to various biotic and abiotic stresses and is believed to be a mechanism of defence against pathogens in plants. This phenomenon is accompanied by increased production of reactive oxygen species (ROS), which can help to destroy invading pathogens. However, the function of chloroplast clustering during viroid infection is unclear. Here, we report that, although the infection by potato spindle tuber viroid (PSTVd) failed to induce chloroplast clustering, chloroplast clustering caused by the overexpression of the *Nicotiana benthamiana* chloroplast outer membrane protein 24 (NbOMP24) promoted the infection by PSTVd, a viroid pathogen, in *N. benthamiana*. Interestingly, H_2_O_2_ treatment, which caused increased ROS accumulation, showed no significant effects on PSTVd infection. Moreover, NbOMP24 protein showed no direct interaction with PSTVd. We propose that perinuclear chloroplast clustering induced by NbOMP24 provides a favourable environment for PSTVd infection. These findings highlight the complexity of chloroplast clustering‐mediated plant–pathogen interactions and the need for further research to fully understand these mechanisms.

## Graphical abstract

Overexpression of outer membrane protein 24 in *Nicotiana benthamiana* induces chloroplast clustering around the nucleus to enhance potato spindle tuber viroid infection.

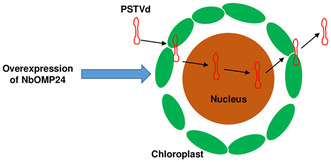

Plants have evolved complex defence mechanisms to protect against various pathogens, including viruses and bacteria. One such mechanism is the clustering of chloroplasts around the nucleus in response to pathogen attack. Chloroplasts are essential organelles that play crucial roles in photosynthesis, carbon fixation and various metabolic processes in plants. In response to stress or pathogen attack, chloroplasts can move toward the nucleus and cluster around it, a process known as chloroplast aggregation or chloroplast migration (Ding et al., 2019; Savage et al., 2021; Zhai et al., 2021). This phenomenon is thought to be a plant defence mechanism that limits the spread of pathogens by creating a physical barrier around the nucleus (Hanson & Conklin, 2020).

Chloroplast clustering around the nucleus is often accompanied by increased production of reactive oxygen species (ROS), which can also contribute to plant defence against pathogens (Torres et al., 2006; Torres & Dangl, 2005; Waszczak et al., 2018). ROS are highly reactive molecules that can damage cellular components, including proteins, lipids and nucleic acids. However, at low concentrations, ROS can also function as signalling molecules that activate defence responses in plants (D'Autréaux & Toledano, 2007; Exposito‐Rodriguez et al., 2017; Gechev et al., 2006; Quan et al., 2008).

Although chloroplast clustering around the nucleus is generally considered a plant defence mechanism (Kumar et al., 2018; Mullineaux et al., 2020; Park et al., 2018), its role in viroid infection has not been investigated. In this study, overexpression of the *Nicotiana benthamiana* chloroplast outer membrane protein 24 (NbOMP24) gene, which promotes chloroplast clustering around the nucleus (Han et al., 2023), was found to enhance the infection of potato spindle tuber viroid (PSTVd), an RNA pathogen that replicates in the nucleus of plant cells (Ding, 2010). Despite not encoding any protein, PSTVd is capable of carrying out biological functions by virtue of its genomic sequence and structural elements that can interact with the appropriate host proteins (Takeda et al., 2011; Wu & Bisaro, 2020; Wu et al., 2019, 2020; Zhong et al., 2006, 2007). Interestingly, H_2_O_2_ treatment, which causes increased ROS accumulation, did not have a significant effect on PSTVd infection, suggesting that the effect of chloroplast clustering on plant–pathogen interactions may be dependent on the specific pathogens involved.

The mechanism behind the effect of chloroplast clustering on PSTVd infection is not fully understood. However, chloroplast clustering might aid PSTVd entry or export from the nucleus by creating a favourable environment, despite the unknown underlying mechanism. This finding reveals that perinuclear chloroplast clustering, a common defence mechanism, plays a role in assisting PSTVd infection and highlights the complexity of plant–pathogen interactions.

Under conditions of stress or pathogen attack, chloroplasts have been observed to relocate toward the nucleus and form clusters around it. This process is known as chloroplast aggregation or chloroplast migration (Ding et al., 2019; Savage et al., 2021; Zhai et al., 2021). To determine whether PSTVd infection can induce chloroplast clustering, the distribution of chloroplasts was analysed in leaves of PSTVd‐infected histone cluster 2‐red fluorescent protein (H2B‐RFP) transgenic plants using a Nikon A1 confocal microscope. (The experimental procedures employed in this study are detailed in File S1.) A control group consisting of H2B‐RFP transgenic plants inoculated with water was included in the study. No significant changes were observed in the distribution of chloroplasts around the nucleus, compared to the control group, in inoculated leaves at 2, 4 and 8 days postinoculation (dpi) or in systemically infected leaves at 20 dpi of PSTVd (Figure 1a,b). These results suggest that PSTVd infection is not capable of inducing chloroplast clustering. It has been reported that *Agrobacterium*‐mediated transient overexpression of the *NbOMP24* gene driven by the CaMV 35S promoter can promote chloroplast clustering around the nucleus in *N*. *benthamiana* cells (Han et al., 2023). Reverse transcription‐quantitative PCR (RT‐qPCR) was performed to assess *NbOMP24* expression in PSTVd‐infected plants at 2, 4, 8 and 20 dpi. The results demonstrated no significant impact of PSTVd infection on *NbOMP24* expression (Figure 1c), consistent with the absence of chloroplast clustering in these plants. In this study, to mimic chloroplast clustering under stress conditions, overexpression of *NbOMP24* was achieved in *N*. *benthamiana* leaves. The transient overexpression of *NbOMP24* was confirmed through western blot analysis of inoculated leaves (Figure S1). Confocal microscopy analysis demonstrated that transient overexpression of *NbOMP24* could induce chloroplast clustering at 2 dpi and this phenomenon persisted for at least 10 dpi (Figure 2a,b). To investigate the potential effects of chloroplast clustering on PSTVd infection, PSTVd rub‐inoculation was performed on leaves with agro‐infiltrated vectors expressing *NbOMP24* or partial β‐glucuronidase (*pGUS*) at 2 dpi. The accumulation of circular form PSTVd (PSTVd‐C), the functional form, was determined in both inoculated and systemic leaves at 8 and 20 dpi, respectively. This analysis comprised a total of seven plants. At 8 dpi of PSTVd infection, the accumulation of PSTVd in *NbOMP24*‐expressing leaves was significantly higher than in *pGUS*‐expressing leaves (Figure 2c,d; *p* < 0.001). The same trend was observed in systemic leaves at 20 dpi (Figure 2e,f; *p* < 0.01). These results suggest that NbOMP24‐induced chloroplast clustering facilitates PSTVd infection. The progeny of PSTVd was analysed by Sanger sequencing in both *NbOMP24*‐expressing and *pGUS*‐expressing leaves. Out of 17 clones sequenced in *NbOMP24*‐expressing leaves, 15 were found to be wild type, while the remaining two clones had A121G and G132C mutations. Similarly, in *pGUS*‐expressing leaves, out of 18 clones sequenced, 16 were wild type, while the remaining two clones had G265A and C144U mutations. Therefore, the induction of chloroplast clustering by *NbOMP24* overexpression did not have any selective effects on the PSTVd sequence population.

**FIGURE 1:**
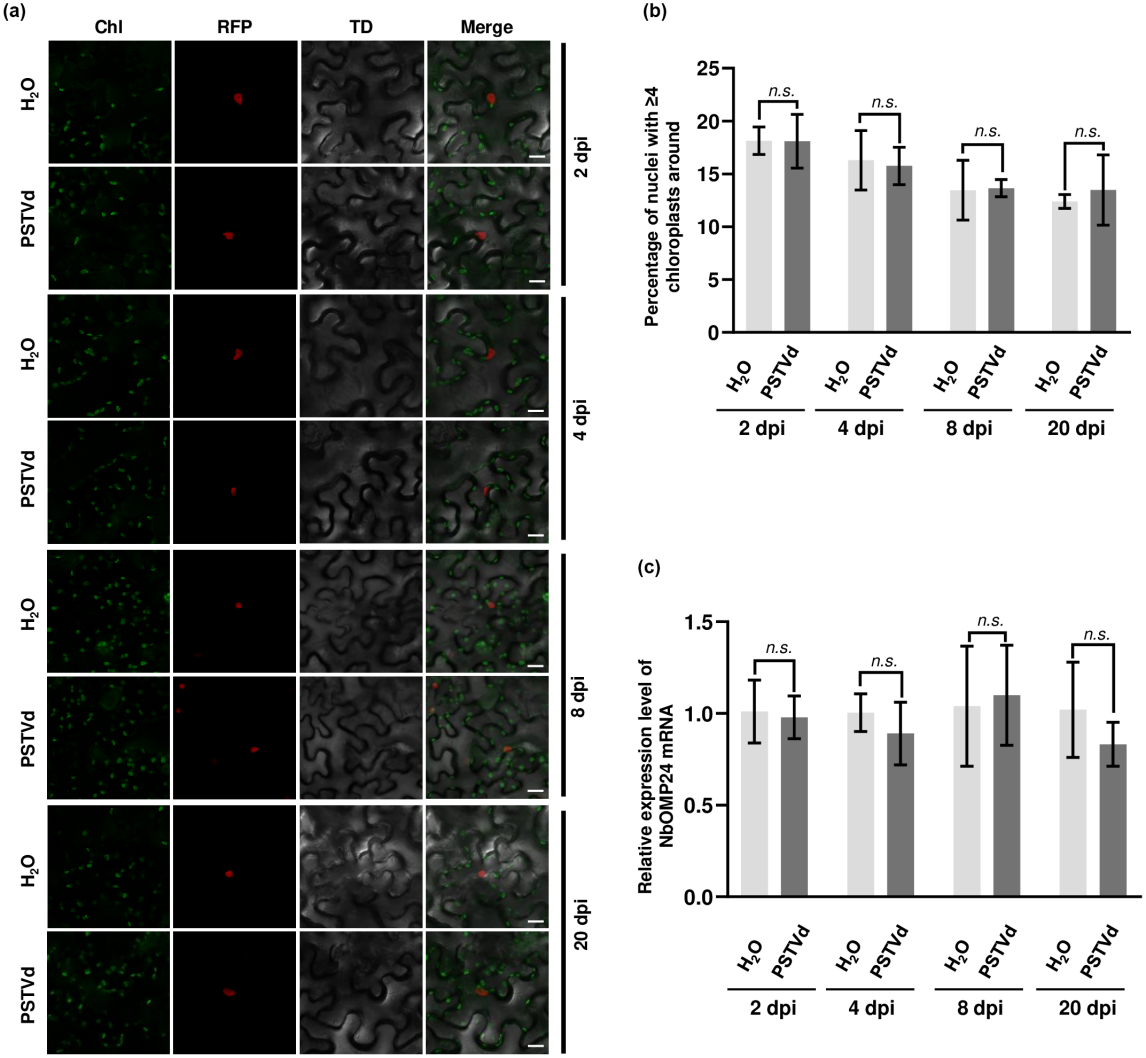
Potato spindle tuber viroid (PSTVd) infection does not induce perinuclear chloroplast clustering. (a) The distribution of chloroplasts was observed under a confocal microscope at 2, 4, 8 and 20 days postinoculation (dpi) of PSTVd infection. Four‐leaf‐stage transgenic *Nicotiana benthamiana* plants expressing histone cluster 2‐red fluorescent protein (H2B‐RFP) were rub‐inoculated with PSTVd. Control plants were inoculated with water. Red fluorescent protein (RFP) fluorescence and chloroplast autofluorescence were visualized using Nikon A1 confocal microscopy. H2B‐RFP served as a nuclear marker. H2B‐RFP fluorescence (RFP, nuclear marker) is in red and chlorophyll autofluorescence (Chl) is in green. TD, transmitted light differential interference contrast. At least 40 cells were observed per sample and representative images are presented. Scale bars 20 μm. (b) Percentage of nuclei with ≥4 chloroplasts surrounding them in PSTVd‐infected plants at 2, 4, 8 and 20 dpi. The raw data are presented in Table S1. n.s., not significant. (c) Reverse transcription‐quantitative PCR analysis of the expression of *NbOMP24* in PSTVd‐infected *N*. *benthamiana* plants at 2, 4, 8 and 20 dpi. *NbUBC* was used as an internal reference gene. n.s., not significant.

**FIGURE 2:**
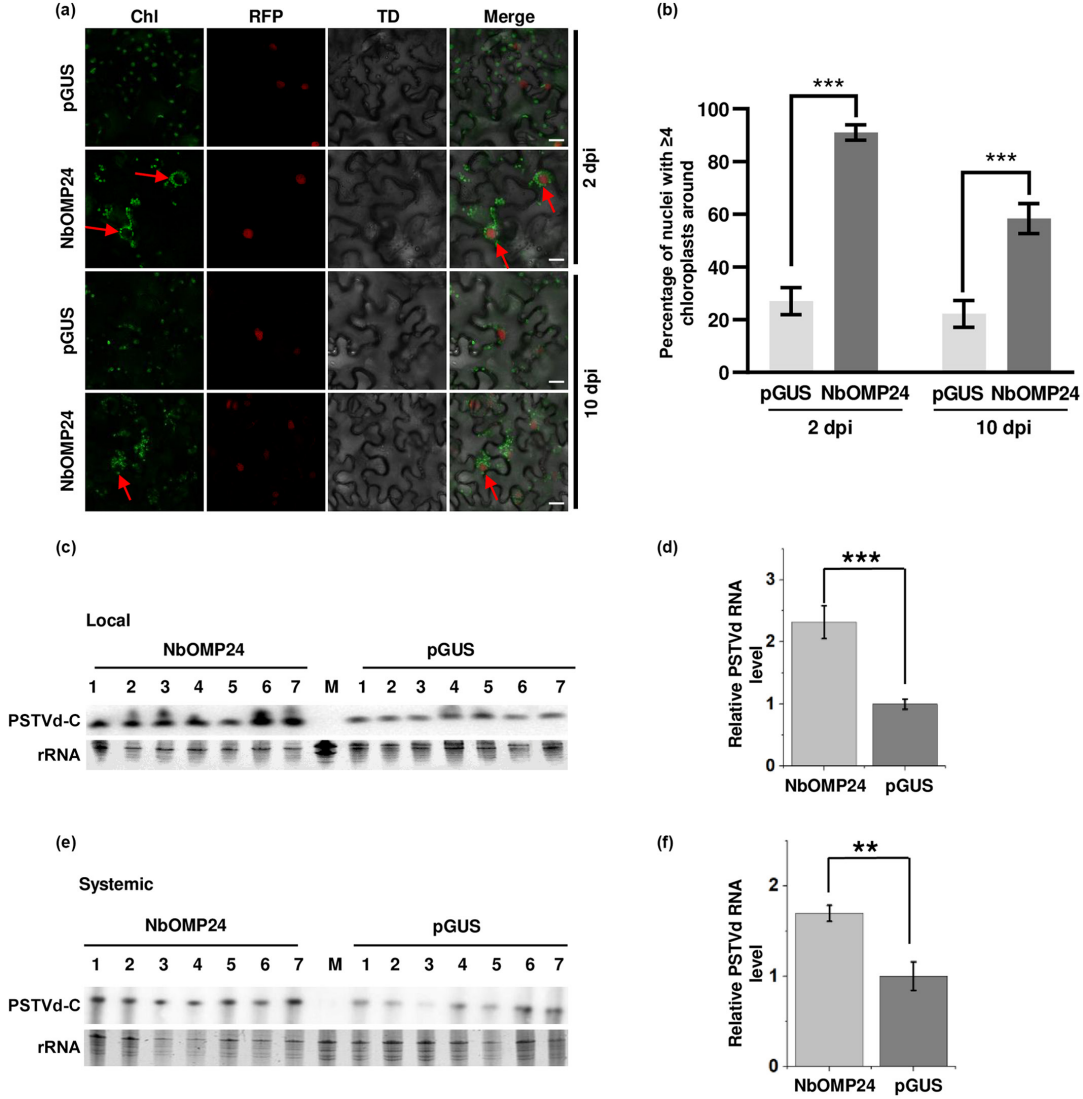
Overexpression of *NbOMP24* facilitated potato spindle tuber viroid (PSTVd) infection. (a) Overexpression of *NbOMP24* resulted in perinuclear chloroplast clustering. *Agrobacterium tumefaciens* GV3101 carrying NbOMP24‐myc or a control (pGUS‐myc) was infiltrated into half of the leaves of H2B‐RFP (histone cluster 2‐red fluorescent protein) transgenic *Nicotiana benthamiana* plants. After 2 or 10 days (dpi), Red fluorescent protein fluorescence and chloroplast autofluorescence (Chl) were visualized using confocal microscopy. TD, transmitted light differential interference contrast. At least 40 cells were observed per sample and representative images are presented. Red arrows indicate the perinuclear chloroplast clustering. Scale bar 20 μm. (b) The percentage of nuclei with ≥4 chloroplasts surrounding them in leaves expressing *NbOMP24* is shown. The raw data can be found in Table S2. ***p ≤ 0.001. (c) At 2 days post‐agro‐infiltration of *Agrobacterium* expressing either *NbOMP24* or *pGUS*, the same leaves were rub‐inoculated with PSTVd. The presence of the functional form PSTVd‐C (circular PSTVd) was examined in the inoculated plants at 8 days postinoculation. GelRed‐stained rRNA was included as a loading control. (d) The signals obtained from RNA blot and GelRed staining presented in (c) were quantified using Quantity One software. The RNA blot signals were normalized to the corresponding GelRed staining signals. The sample with the lowest value was set as the reference with a value of 1, and other samples were normalized to this reference value. Data were compared by two‐tailed unpaired *t* test, ***p < 0.001. (e) The same method as described in (c) was used to detect systemic PSTVd‐C infection. (f) The same method as described in (d) was employed to quantify the data presented in (c), **p < 0.01.

In summary, it was found that unlike plant viruses, such as turnip mosaic virus (Zhai et al., 2021), tomato yellow leaf curl virus, beet curly top virus, abutilon mosaic virus and tobacco mosaic virus (TMV) (Ding et al., 2019), PSTVd infection was unable to induce perinuclear chloroplast clustering during its whole infection stage. Moreover, it was observed that NbOMP24‐mediated chloroplast clustering unexpectedly promoted PSTVd infection.

A significant role of ROS burst in pathogen infection has been documented in previous research (Ishiga et al., 2012; Torres et al., 2006; Waszczak et al., 2018; Wu et al., 2017; Zurbriggen et al., 2009). ROS serve as signalling molecules, facilitating organelle‐to‐organelle and organelle‐to‐nucleus communication. They regulate various plant processes under normal and stress conditions (Ishiga et al., 2017; Mittler et al., 2022; Sandalio et al., 2021). Perinuclear chloroplast clustering plays a crucial role in facilitating the transfer of chloroplast retrograde signals, including ROS, to the nucleus. This process leads to the upregulation of resistance gene expression, thereby enhancing plant immunity (Hanson & Conklin, 2020; Hanson & Hines, 2018; Mullineaux et al., 2020). In our earlier study, perinuclear chloroplast clustering mediated by NbOMP24 was found to promote the accumulation of ROS (Han et al., 2023). In the present study, *NbOMP24*‐expressing leaves exhibited higher H_2_O_2_ content than the control leaves at 2 dpi. However, at 10 dpi, despite the persistence of perinuclear chloroplast clustering induced by *NbOMP24* expression, the H_2_O_2_ content was comparable to that of the control (Figure 3a). It is known that the ROS burst can exert an antiviral effect; therefore, it is important to determine whether ROS play a role in PSTVd infection. To investigate this, six *N*. *benthamiana* plants were subjected to a 10 mM H_2_O_2_ spray treatment. Staining with nitroblue tetrazolium chloride (NBT) (Figure 3b) and 3,3′‐diaminobenzidine (DAB) (Figure 3c) was performed 1 and 3 h later. Both assays demonstrated that the H_2_O_2_ treatment induced ROS burst at both time points. Consistently, RT‐qPCR results showed the mRNA levels of the H_2_O_2_‐responsive gene *N*. *benthamiana catalase 1* (*NbCAT1*) (Garcia‐Brugger et al., 2006) were increased at both time points after H_2_O_2_ treatment (Figure 3d). However, confocal microscopy analysis revealed that H_2_O_2_ treatment did not significantly induce perinuclear chloroplast clustering in H2B‐RFP plants at 1 or 3 h after spraying (Figure 3e,f and Table S3). The current finding contradicts a prior study that suggested that chloroplast clustering could be induced by the exogenous application of 10 μM H_2_O_2_. This discrepancy could be attributed to the fact that this study used H2B transgenic plants to label the nucleus, whereas transient green fluorescent protein (GFP) expression was employed in the previous study (Ding et al., 2019). Another possibility is the method of treatment employed. In this study, H_2_O_2_ was sprayed onto the surface of the leaves, whereas the previous study involved the infiltration of H_2_O_2_. PSTVd rub‐inoculation was executed 1 or 3 h after H_2_O_2_ treatment. Therefore, this methodology can be used to investigate the function of ROS bursts in PSTVd infection by eliminating the impact of perinuclear chloroplast clustering. The accumulation of PSTVd‐C was assessed at 8 and 20 dpi, respectively. In comparison to the water‐treated plants, the accumulation of PSTVd‐C was not significantly affected by H_2_O_2_ treatment in both inoculated leaves and systemic leaves (Figure 3g–j). Our data revealed that ROS at the early stage of PSTVd infection has no significant effects on its RNA accumulation. However, it is worth noting that H_2_O_2_ treatment cannot be directly compared to the sustained ROS induction achieved with the overexpression of *NbOMP24*.

**FIGURE 3:**
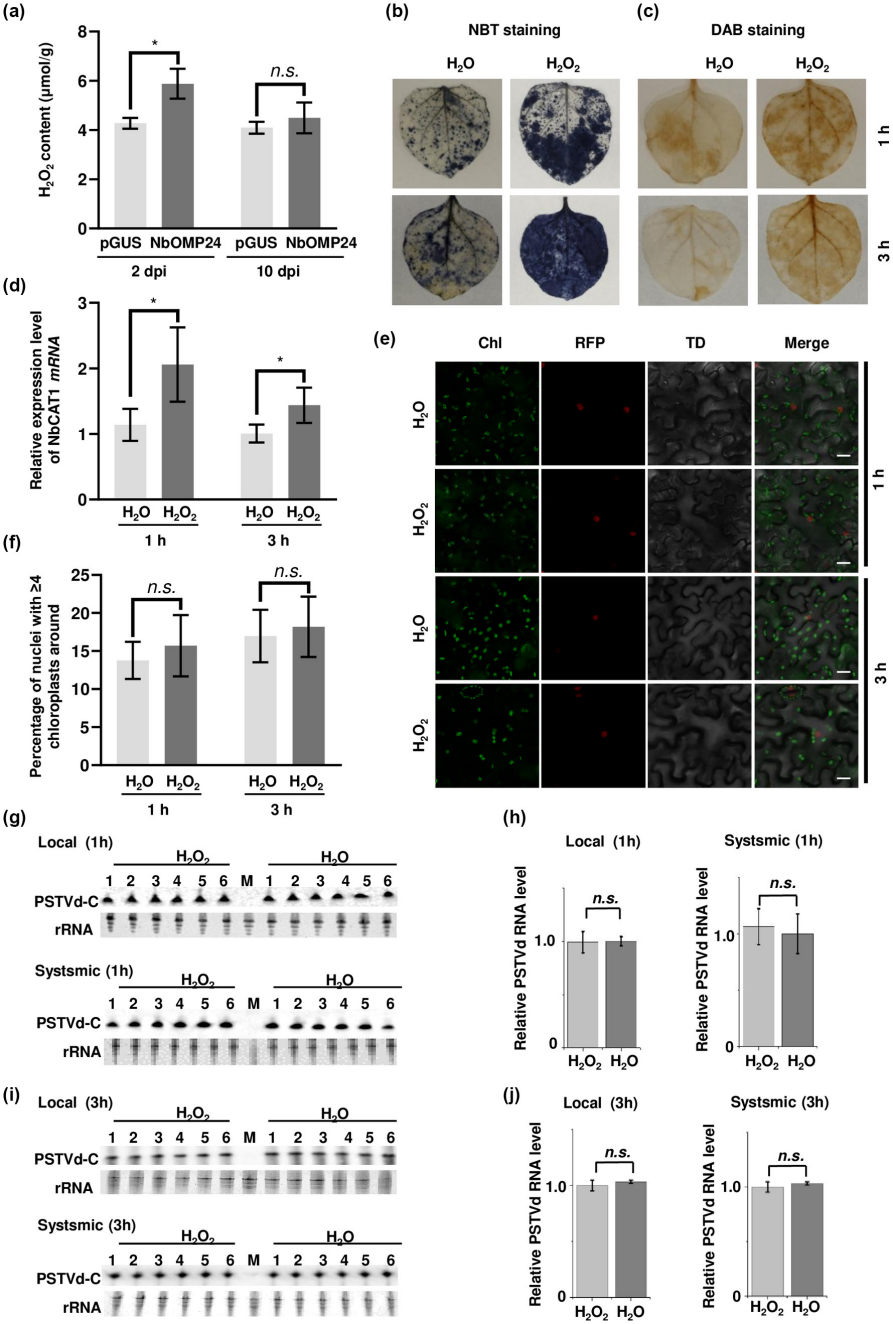
Exogenous application of H_2_O_2_ showed no significant effect on potato spindle tuber viroid (PSTVd) infection. (a) H_2_O_2_ content in *NbOMP24‐* or *pGUS*‐expressing leaves were determined at 2 and 10 days postinoculation (dpi). The H_2_O_2_ content in the samples was quantified using a spectrophotometric method, based on the reaction of H_2_O_2_ with titanium sulphate to form a yellow titanium peroxide complex with a characteristic absorption at 415 nm. *p < 0.05; n.s., not significant. (b) Exogenous application of H_2_O_2_ resulted in reactive oxygen species (ROS) burst. *Nicotiana benthamiana* plants were subjected to H_2_O_2_ (10 mM) spray treatment for 1 or 3 h. The ROS burst was detected by nitroblue tetrazolium chloride (NBT) staining. (c) The ROS burst was confirmed by 3,3′‐diaminobenzidine (DAB) staining. (d) Reverse transcription‐quantitative PCR analysis of the expression of H_2_O_2_‐responsive gene, *catalase 1* (*CAT1*), at mRNA level. *p < 0.05. (e) Confocal microscopy analysis of chloroplast distribution in leaves of H2B‐RFP (histone cluster 2‐red fluorescent protein) plants with H_2_O_2_ or water treatment. *N*. *benthamiana* plants were first treated with exogenous H_2_O_2_ (10 mM). The distribution of chloroplasts was observed under a confocal microscope Nikon A1 1 and 3 h after H_2_O_2_ or water treatment. Chl, chloroplast autofluorescence; TD, transmitted light differential interfence contrast. (f) The percentage of nuclei with ≥4 chloroplasts surrounding them in leaves treated with H_2_O_2_ or water is presented. The raw data can be found in Table S1. n.s., not significant. (g) PSTVd rub‐inoculation was executed 1 h after H_2_O_2_ treatment. The accumulation of PSTVd‐C (circular PSTVd) in inoculated leaves and systemic leaves was analysed at 8 or 20 dpi. GelRed‐stained rRNA was included as the loading control. (h) The signals from the RNA blot and GelRed staining presented in (g) were quantified using Quantity One software. The RNA blot signals were normalized to the corresponding GelRed staining signals. The sample with the lowest value was set as the reference value of 1, and other samples were normalized to this reference value. n.s., not significant. (i) The same method as described in (g) was used to detect the accumulation of PSTVd‐C 3 h after H_2_O_2_ treatment. (j) The same method as described in (h) was used to quantify the data presented in (i). n.s., not significant.

To investigate the mechanism underlying the role of NbOMP24 in PSTVd infection, RNA binding protein immunoprecipitation (RIP) was conducted to test the potential binding of NbOMP24 to PSTVd. In brief, rub‐inoculation of PSTVd was performed on 3‐week‐old *N*. *benthamiana* plants. Transient expression of *NbOMP24* was achieved by agro‐infiltration of a vector expressing *NbOMP24* into the inoculated leaves. Transient expression of *pGUS*, which served as a negative control, and TMV movement protein (*MP*), which served as a positive control (Wu & Bisaro, 2022), were also performed by agro‐infiltration. The expression of NbOMP24, pGUS, and TMV MP was confirmed by western blotting 3 days after agro‐infiltration (Figure 4a). RNA was extracted from the RIP samples using TRIzol reagent, followed by RT‐PCR to detect the presence of PSTVd. Results of the RIP assay indicated that TMV MP could bind PSTVd, while neither NbOMP24 nor pGUS was found to bind PSTVd (Figure 4b). Therefore, it is unlikely that the role of NbOMP24 in PSTVd infection is achieved through direct binding.

**FIGURE 4:**
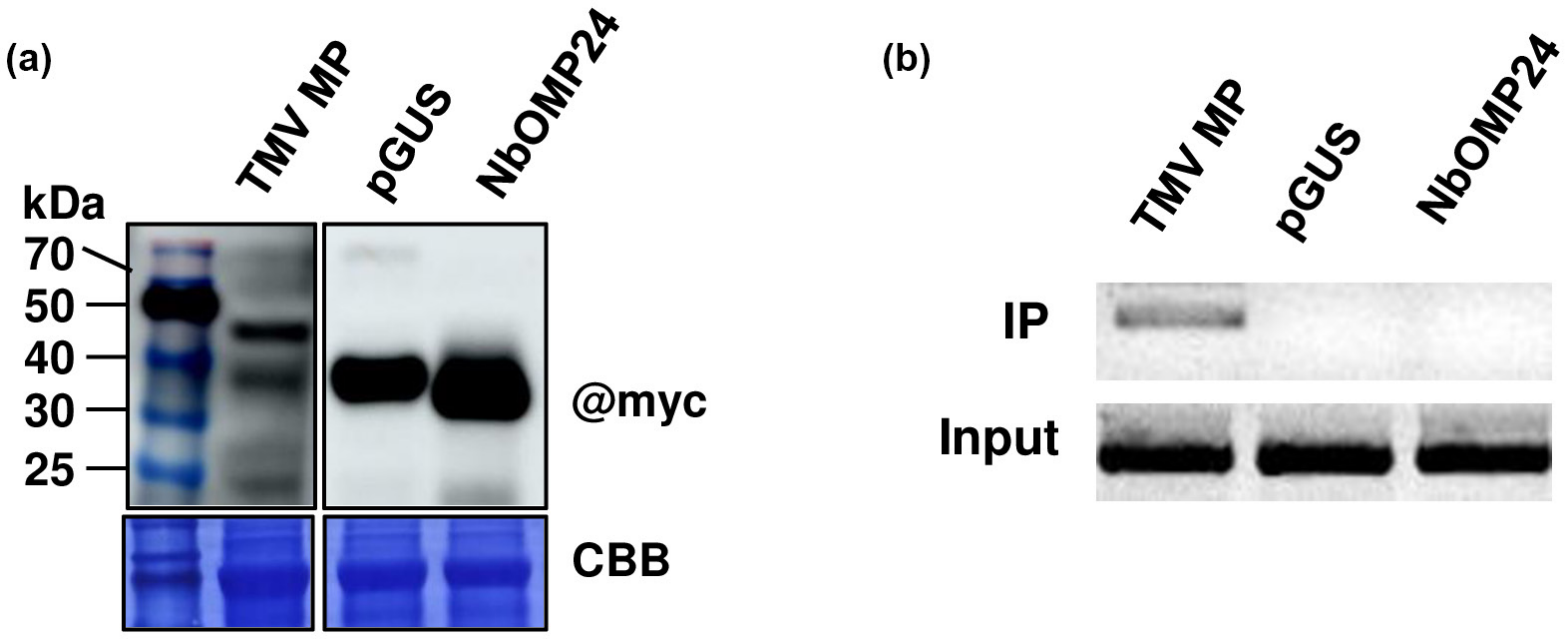
*Nicotiana benthamiana* chloroplast outer membrane protein 24 (NbOMP24) was unable to bind to potato spindle tuber viroid (PSTVd). (a) The expression of NbOMP24, pGUS and TMV movement protein (MP) was analysed by western blot. *Nicotiana benthamiana* leaves were rub‐inoculated with PSTVd transcripts, followed by agro‐infiltration with *NbOMP24*, *pGUS* and TMV *MP* 5 days later. Inoculated leaves were collected 3 days later and analysed using an anti‐Myc antibody. Coomassie brilliant blue (CBB)‐stained RbcL was used as the loading control. (b) RNA binding protein immunoprecipitation (RIP) assay was conducted using Myc‐Trap magnetic agarose beads to identify any potential binding between NbOMP24 and PSTVd. RNA was extracted from the RIP samples (Input) and immunoprecipitation beads (IP) using TRIzol reagent, and subsequently subjected to reverse transcription (RT)‐PCR analysis to detect the presence of PSTVd. RT‐PCR applied to detect PSTVd was conducted for 26 cycles.

The finding that chloroplast clustering around the nucleus may not always have a protective effect against pathogens raises questions about the mechanism underlying the effect of chloroplast positioning on plant–pathogen interactions. It is known that chloroplasts can produce ROS, which can act as signalling molecules to activate plant defence responses (Carr et al., 2019; Hernández et al., 2016). However, ROS can also be detrimental to the plant by causing oxidative damage to cellular components (Shafi et al., 2022). Therefore, the relationship between chloroplast clustering and ROS production is complex, and the effect of chloroplast positioning on plant–pathogen interactions may be contingent on the specific type of pathogens involved.

Chloroplast clustering around the nucleus is a recognized response mechanism to different biotic and abiotic stresses, which is commonly regarded as a plant defence mechanism against pathogens (Kumar et al., 2018; Mullineaux et al., 2020; Park et al., 2018). For example, chloroplast clustering has been reported to be a defence mechanism against bacterial pathogens such as *Pseudomonas syringae* (Ding et al., 2019). However, it is currently unknown whether chloroplast clustering plays a role in the infection of viroids, a significant group of plant pathogens. In the case of PSTVd, we showed that chloroplast clustering around the nucleus may provide a favourable environment for PSTVd infection, while the underlying mechanism is unknown. This hypothesis is supported by previous studies showing that chloroplasts can interact with the nucleus and affect gene expression (Exposito‐Rodriguez et al., 2017; Song et al., 2021). Furthermore, it has been reported that chloroplasts can form physical associations with the nucleus through membrane extensions, suggesting that chloroplasts may be involved in intracellular communication (Navarro et al., 2021).

Another factor that may contribute to the context‐dependent nature of chloroplast clustering is the signalling pathways involved. It has been reported that different signalling pathways may be activated in response to different types of stress, including pathogen attacks (Fujita et al., 2006). For example, the salicylic acid signalling pathway is important for defence against biotrophic pathogens (Boatwright & Pajerowska‐Mukhtar, 2013), whereas the jasmonic acid signalling pathway is involved in defence against necrotrophic pathogens (Ghozlan et al., 2020). It is possible that the effect of chloroplast clustering on plant–pathogen interactions is mediated by different signalling pathways depending on the type of pathogen.

In conclusion, unlike some viruses, the results here demonstrate that perinuclear chloroplast clustering is not induced by PSTVd and has a positive role in assisting its infection with an unknown mechanism. The effect of chloroplast positioning on plant–pathogen interactions is likely to be context‐dependent and influenced by various factors, including the type of pathogen, the stage of infection, the plant species and the signalling pathways involved. Further research is needed to elucidate the molecular mechanisms underlying the effect of chloroplast clustering on plant–pathogen interactions and to identify the factors that determine the context‐dependent nature of this phenomenon.

## CONFLICT OF INTEREST STATEMENT

The authors declare that they have no conflict of interest.

## Supporting information


**FILE S1** Experimental procedures.

**FIGURE S1** Confirmation of *NbOMP24* overexpression by western blot. Western blot analysis was used to detect the expression of NbOMP24 at 2 days postinoculation in Figure 2a. Coomassie brilliant blue‐stained RbcL was included as the loading control.

**TABLE S1** Raw data of the chloroplast counts presented in Figure 1b. The *p* values were calculated using an unpaired *t* test to compare the percentage of nuclei with ≥4 chloroplasts around in PSTVd‐infected leaves with the corresponding water control

**TABLE S2** Raw data of the chloroplast counts presented in Figure 1d. The *p* values were calculated using an unpaired *t* test to compare the percentage of nuclei with ≥4 chloroplasts around in NbOMP24‐infiltrated leaves with the corresponding pGUS control

**TABLE S3** Raw data of the chloroplast counts presented in Figure 2f. *p* values were calculated using an unpaired *t* test to compare the percentage of nuclei with ≥4 chloroplasts around in H_2_O_2_‐treated leaves with the corresponding water control

## Data Availability

The data that support the findings of this study are available from the corresponding author upon reasonable request.
