# Supplementary figures and images for: Chloroplast clustering around the nucleus induced by OMP24 overexpression unexpectedly promoted PSTVd infection in Nicotiana benthamiana
Source: Mol Plant Pathol. 2023 Sep 11;24(12):1552–9. doi: 10.1111/mpp.13385 (PMC10632781; doi:10.1111/mpp.13385)

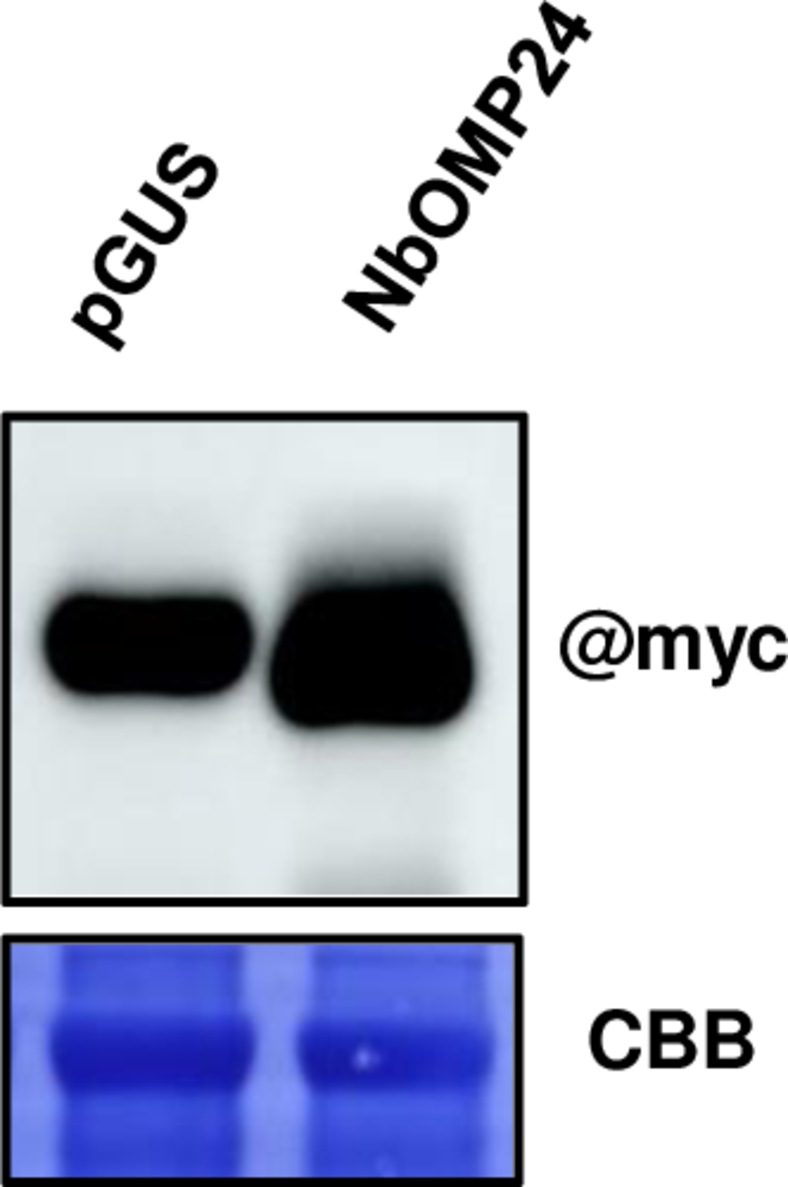

Supplement: Supplementary file 1 — FILE S1 Experimental procedures. [file MPP-24-1552-s005.tif]
