# Supplementary material for: Chloroplast clustering around the nucleus induced by OMP24 overexpression unexpectedly promoted PSTVd infection in Nicotiana benthamiana
Source: Mol Plant Pathol. 2023 Sep 11;24(12):1552–9. doi: 10.1111/mpp.13385 (PMC10632781; doi:10.1111/mpp.13385)
Supplement: Supplementary file 2 — FIGURE S1 Confirmation of NbOMP24 overexpression by western blot. Western blot analysis was used to detect the expression of NbOMP24 at 2 days postinoculation in Figure 2a. Coomassie brilliant blue‐stained RbcL was included as the loading control. [file MPP-24-1552-s003.docx]

**EXPERIMENTAL PROCEDURES**

**Plants and agroinoculation**

Wild-type and H2B-RFP transgenic *Nicotiana benthamiana* plants were cultivated in greenhouse of Ningbo University. *Agrobacterium tumefaciens* was used to transfect pCAMBIA1301 vectors expressing NbOMP24-myc, pGUS-myc and TMV MP-myc. Agrobacteria were cultivated in LB medium supplemented with 25 µg/mL kanamycin and 100 µg/mL rifampicin at 28°C overnight. The following day, the agrobacteria were resuspended in an MMA solution (10 mM MgCl_2_, 100 µM acetosyringone, 10 mM MES pH 5.6). OD_600_ values were adjusted to 0.5 for inoculation. Agroinoculation was performed on the first two true leaves of seven 2-week-old *N. benthamiana* plants.

**H_2_O_2_ treatment**

H_2_O_2_ solution (10 mM in ddH_2_O) was sprayed onto the first two true leaves of six 2-week-old *N. benthamiana* plants. To perform the control experiment, the first two true leaves of another six 2-week-old *N. benthamiana* plants were sprayed with the same amount of ddH_2_O.

**PSTVd inoculation**

PSTVd infection was performed by rub-inoculating the PSTVd inoculum onto the surface of *N. benthamiana*, which had been previously coated with carborundum powder, either 2 days after agroinoculation of pCAMBIA1301 vectors expressing NbOMP24-myc and pGUS-myc at 1 or 3 h after H_2_O_2_ treatment. The inoculum was made up of PSTVd (+)-strand *in vitro* transcripts (450 ng/plant) dissolved in diethylpyrocarbonate (DEPC)-treated water. Inoculation of DEPC-treated water also served as a mock inoculation.

**RNA isolation and RNA blot**

Inoculated leaves and systemic leaves were sampled at 8 and 20 days after inoculation (dpi) of PSTVd. Plant RNA samples were prepared using TRIzol reagent (Invitrogen). Prior to further experiments, DNase I (Invitrogen) was used to incubate RNA samples at 37℃ for 2 h to eliminate genomic DNA. RNA blotting was conducted using a DIG-labelled probe (Wu & Bisaro, 2022). The preparation of (DIG)-labelled PSTVd riboprobes involved *in vitro* transcription, where the pInter(−) plasmid was linearized with SpeI (New England Biolabs), and the MAXIscript T7 transcription kit (ThermoFisher Scientific) was used. Signal capture was carried out using Amersham Imager 600 (GE Healthcare Life Sciences).

**RT-PCR and PSTVd progeny sequencing**

Reverse transcription (RT) was performed using RNA samples as a template and Superscript III reverse transcriptase (ThermoFisher Scientific) as the reagent. The primer used in RT was PSTVd86R (5’-TGAAGCGCTCCTCCGAGCC-3’). Full-length progeny sequences were amplified from reverse transcription products that were greater than full-length, following the previously described procedure ([Wu et al., 2020](#_ENREF_29)). PSTVd86F (5’-GGGATCCCCGGGGAAACC-3’) and PSTVd86R were used as the forward and reverse primers, respectively. The RT-PCR amplified progeny products were purified using the Zymoclean Gel DNA Recovery Kit (Zymo Research) after agarose gel electrophoresis. Subsequently, purified products were cloned into PCR2.1 with a TA Cloning Kit (Invitrogen). DNA sequencing was performed at Zhejiang Youkang Biotechnology Co., Ltd.

**RT-qPCR assay**

Total RNA samples were prepared using TRIzol reagent (Invitrogen) and were used as a template to prepare cDNA through reverse transcription using the ReverTra Ace qPCR RT Master Mix with gDNA Remover Kit from Toyobo. qPCR mix was prepared using SYBR Green Master Mix kit, and all reactions were performed on a LightCycler 384 from Roche Applied Science.

To determine the expression of *NbOMP24*, leaves were collected at 2, 4, 8 20 dpi of PSTVd infection, while water-treated plants served as the control. To measure the expression level of *NbCAT1*, leaves were collected at 1 or 3 h after H_2_O_2_ treatment. The primers used to detect *NbOMP24* and *NbCAT*1 were *NbOMP24*-qRT-F (5’- TGAACCCGATACTAACCCCG-3’), *NbOMP24*-qRT-R (5’-GAGGATCGTCATTGGATGGC-3’)*, NbCAT1*-qRT-F (5’-TATGCACAGGGACGA GGAGA-3’) and *NbCAT1*-qRT-R (5’-TGGTGTGAATGAGCGGTACC-3’). *NbUBC* was used as the reference gene (Zhang *et al.*, 2023), and data were analysed using the 2^−∆∆Ct^ method.

**Western blot**

Protein extraction from leaf tissues was performed 2 days after agro-inoculation, and the total proteins were separated by 12% SDS-PAGE. The separated proteins were then transferred onto an Immobilon-P PVDF membrane from Millipore, Merck. Protein detection was performed using an anti-Myc antibody from TransGen Biotech and visualized using Immobilon western HRP substrate from Millipore. To ensure equal protein loading, Coomassie Brilliant Blue staining RBCL was used as a loading control.

**Analysis of ROS accumulation by DAB and NTB staining**

Histochemical detection of H_2_O_2_ and superoxide in leaves expressing NbOMP24-myc or pGUS-myc was performed using 3,3'-diaminobenzidine (DAB, MCE) and nitrotetrazolium blue chloride (NBT, MCE) staining, respectively (Qiao et al., 2015).

**Measurement of H_2_O_2_ concentration**

The concentration of H_2_O_2_ was determined using an H_2_O_2_ reagent kit (Comin Biotechnology, Suzhou, China) following the manufacturer's instructions. The kit uses a yellow titanium peroxide complex, which is formed by the reaction of H_2_O_2_ and titanium sulphate and exhibits a characteristic absorption at 415 nm. The absorbance of NbOMP24 or pGUS-expressed samples at 415 nm was measured using a microplate reader (BioTek) to quantify the H_2_O_2_ content.

**RNA Binding Protein Immunoprecipitation (RIP) assay**

Transient expression of TMV MP, NbOMP24, and pGUS was carried out through agro-inoculation 5 days after PSTVd inoculation. Three days after agro-inoculation, leaves were collected and cross-linked in ice-cold PBS buffer at a dose of 0.4 J/cm^2^. RIP lysate was extracted using pre-cooled RIP buffer (150 mM KCl, 25 mM Tris-HCl pH 7.4, 5 mM EDTA, 0.5 mM DTT, 0.5% NP-40, 100 U/mL RNase inhibitor, and 1 × protease inhibitor cocktail) followed by immunoprecipitation using Myc-Trap Magnetic Agarose beads (Chromotek) for 3–4 h at 4°C. The beads were then collected using DynaMag Magnets (Invitrogen) and washed 5–7 times with RIP buffer. RNA was extracted from the beads and used for RT-PCR to detect PSTVd.

**REFERENCES**

Qiao, B., Zhang, Q., Liu, D., Wang, H., Yin, J., Wang, R., He, M., Cui, M., Shang, Z., Wang, D., & Zhu, Z. (2015). A calcium-binding protein, rice annexin OsANN1, enhances heat stress tolerance by modulating the production of H_2_O_2_. *Journal of Experimental Botany*, **66**, 5853–5866.

Wu, J. and Bisaro, D. M. (2022) Tobacco mosaic virus movement protein complements a potato spindle tuber viroid RNA mutant impaired for mesophyll entry but not mutants unable to enter the phloem. *PLoS Pathogens,* **18,** e1011062.

Wu, J., Zhou, C., Li, J., Li, C., Tao, X., Leontis, N. B., et al. (2020) Functional analysis reveals G/U pairs critical for replication and trafficking of an infectious non-coding viroid RNA. *Nucleic Acids Research*, **48**, 3134–3155.

Zhang, G., Zhang, Z., Wan, Q., Zhou, H., Jiao, M., Zheng, H.*,* et al*.* (2023) Selection and validation of reference genes for RT-qPCR analysis of gene expression in *Nicotiana* *benthamiana* upon single infections by 11 positive-sense single-stranded RNA viruses from four genera. *Plants (Basel),* **12** ,857.
