# Supplementary material for: Chloroplast clustering around the nucleus induced by OMP24 overexpression unexpectedly promoted PSTVd infection in Nicotiana benthamiana
Source: Mol Plant Pathol. 2023 Sep 11;24(12):1552–9. doi: 10.1111/mpp.13385 (PMC10632781; doi:10.1111/mpp.13385)
Supplement: Supplementary file 3 — TABLE S1 Raw data of the chloroplast counts presented in Figure 1b. The p values were calculated using an unpaired t test to compare the percentage of nuclei with ≥4 chloroplasts around in PSTVd‐infected leaves with the corresponding water control [file MPP-24-1552-s002.docx]

**Supplementary Table S1: Raw data of the chloroplast counts presented in Figure 1B.** P values were calculated using an unpaired *t-test* to compare the percentage of nuclei with ≥4 chloroplasts around in PSTVd-infected leaves with the corresponding H_2_O control.

| Repeats | Samples | Number of nuclei checked | Number of nuclei with ≥4 chloroplasts around | Percentage of nuclei with ≥4 chloroplasts around | STDEV.P |
| --- | --- | --- | --- | --- | --- |
| Repeat 1 | H2O 2dpi | 42 | 7 | 16.67 | 0.491337472 |
|  | PSTVd 2dpi | 45 | 9 | 20 |  |
| Repeat 2 | H2O 2dpi | 43 | 8 | 18.6 |  |
|  | PSTVd 2dpi | 42 | 8 | 19.05 |  |
| Repeat 3 | H2O 2dpi | 47 | 9 | 19.15 |  |
|  | PSTVd 2dpi | 46 | 7 | 15.22 |  |
| Repeat 1 | H2O 4dpi | 41 | 8 | 19.51 | 0.418675376 |
|  | PSTVd 4dpi | 47 | 7 | 14.89 |  |
| Repeat 2 | H2O 4dpi | 49 | 7 | 14.29 |  |
|  | PSTVd 4dpi | 45 | 8 | 17.78 |  |
| Repeat 3 | H2O 2dpi | 53 | 8 | 15.09 |  |
|  | PSTVd 4dpi | 48 | 7 | 14.58 |  |
| Repeat 1 | H2O 8dpi | 47 | 5 | 10.64 | 0.457681688 |
|  | PSTVd 8dpi | 46 | 6 | 13.04 |  |
| Repeat 2 | H2O 8dpi | 43 | 7 | 16.28 |  |
|  | PSTVd 8dpi | 45 | 6 | 13.33 |  |
| Repeat 3 | H2O 8dpi | 52 | 7 | 13.46 |  |
|  | PSTVd 8dpi | 48 | 7 | 14.58 |  |
| Repeat 1 | H2O 20dpi | 47 | 6 | 12.77 | 0.301112969 |
|  | PSTVd 20dpi | 44 | 5 | 11.36 |  |
| Repeat 2 | H2O 20dpi | 43 | 5 | 11.63 |  |
|  | PSTVd 20dpi | 51 | 6 | 11.76 |  |
| Repeat 3 | H2O 20dpi | 47 | 6 | 12.77 |  |
|  | PSTVd 20dpi | 52 | 9 | 17.31 |  |
