# Supplementary material for: Chloroplast clustering around the nucleus induced by OMP24 overexpression unexpectedly promoted PSTVd infection in Nicotiana benthamiana
Source: Mol Plant Pathol. 2023 Sep 11;24(12):1552–9. doi: 10.1111/mpp.13385 (PMC10632781; doi:10.1111/mpp.13385)
Supplement: Supplementary file 4 — TABLE S2 Raw data of the chloroplast counts presented in Figure 1d. The p values were calculated using an unpaired t test to compare the percentage of nuclei with ≥4 chloroplasts around in NbOMP24‐infiltrated leaves with the corresponding pGUS control [file MPP-24-1552-s001.docx]

**Supplementary Table S2: Raw data of the chloroplast counts presented in Figure 1D.** P-values were calculated using an unpaired *t-test* to compare the percentage of nuclei with ≥4 chloroplasts around in NbOMP24-infiltrated leaves with the corresponding pGUS control.

| Repeats | Samples | Number of nuclei checked | Number of nuclei with ≥4 chloroplasts around | Percentage of nuclei with ≥4 chloroplasts around | STDEV.P |
| --- | --- | --- | --- | --- | --- |
| Repeat 1 | pGUS 2dpi | 47 | 13 | 27.66 | 0.000956937 |
|  | NbOMP24 2dpi | 42 | 37 | 88.10 |  |
| Repeat 2 | pGUS 2dpi | 51 | 11 | 21.57 |  |
|  | NbOMP24 2dpi | 45 | 41 | 91.11 |  |
| Repeat 3 | pGUS 2dpi | 44 | 14 | 31.82 |  |
|  | NbOMP24 2dpi | 49 | 46 | 93.88 |  |
| Repeat 1 | pGUS 10dpi | 43 | 10 | 23.26 | 0.007431805 |
|  | NbOMP24 10dpi | 52 | 27 | 51.92 |  |
| Repeat 2 | pGUS 10dpi | 48 | 8 | 16.67 |  |
|  | NbOMP24 10dpi | 51 | 31 | 60.78 |  |
| Repeat 3 | pGUS 10dpi | 45 | 12 | 26.67 |  |
|  | NbOMP24 10dpi | 48 | 30 | 62.50 |  |
