# Supplementary material for: Chloroplast clustering around the nucleus induced by OMP24 overexpression unexpectedly promoted PSTVd infection in Nicotiana benthamiana
Source: Mol Plant Pathol. 2023 Sep 11;24(12):1552–9. doi: 10.1111/mpp.13385 (PMC10632781; doi:10.1111/mpp.13385)
Supplement: Supplementary file 5 — TABLE S3 Raw data of the chloroplast counts presented in Figure 2f. p values were calculated using an unpaired t test to compare the percentage of nuclei with ≥4 chloroplasts around in H2O2‐treated leaves with the corresponding water control [file MPP-24-1552-s004.docx]

**Supplementary Table S3: Raw data of the chloroplast counts presented in Figure 2F.** P values were calculated using an unpaired *t-test* to compare the percentage of nuclei with ≥4 chloroplasts around in H_2_O_2_-treated leaves with the corresponding H_2_O control.

|  | Samples | Number of nuclei checked | Number of nuclei with ≥4 chloroplasts around | Percentage of nuclei with ≥4 chloroplasts around | STDEV.P |
| --- | --- | --- | --- | --- | --- |
| Repeat 1 | H_2_O 1h | 44 | 7 | 15.91 | 0.326925293 |
|  | H_2_O_2_ 1h | 52 | 6 | 11.54 |  |
| Repeat 2 | H_2_O 1h | 42 | 6 | 14.29 |  |
|  | H_2_O_2_ 1h | 50 | 8 | 16.00 |  |
| Repeat 3 | H_2_O 1h | 45 | 5 | 11.11 |  |
|  | H_2_O_2_ 1h | 46 | 9 | 19.57 |  |
| Repeat 1 | H_2_O 3h | 43 | 9 | 20.93 | 0.399666651 |
|  | H_2_O_2_ 3h | 51 | 7 | 13.73 |  |
| Repeat 2 | H_2_O 3h | 52 | 8 | 15.38 |  |
|  | H_2_O_2_ 3h | 47 | 10 | 21.28 |  |
| Repeat 3 | H_2_O 3h | 48 | 7 | 14.58 |  |
|  | H_2_O_2_ 3h | 46 | 9 | 19.57 |  |
